# Association of Arachidonic Acid-derived Lipid Mediators with Subsequent Onset of Acute Myocardial Infarction in Patients with Coronary Artery Disease

**DOI:** 10.1038/s41598-020-65014-z

**Published:** 2020-05-15

**Authors:** Chin-Chou Huang, Meng-Ting Chang, Hsin-Bang Leu, Wei-Hsian Yin, Wei-Kung Tseng, Yen-Wen Wu, Tsung-Hsien Lin, Hung-I Yeh, Kuan-Cheng Chang, Ji-Hung Wang, Chau-Chung Wu, Lie-Fen Shyur, Jaw-Wen Chen

**Affiliations:** 1 Department of Medical Education, Taipei Veterans General Hospital, Taipei, Taiwan; 2 Division of Cardiology, Department of Medicine, Taipei Veterans General Hospital, Taipei, Taiwan; 3 Cardiovascular Research Center, National Yang-Ming University, Taipei, Taiwan; 4 Institute of Pharmacology, National Yang-Ming University, Taipei, Taiwan; 5 Agricultural Biotechnology Research Center, Academia Sinica, Taipei, Taiwan; 6 Department of Biochemical Science and Technology, College of Life Science, National Taiwan University, Taipei, Taiwan; 7 Healthcare and Service Center, Taipei Veterans General Hospital, Taipei, Taiwan; 8 Institute of Clinical Medicine, National Yang-Ming University, Taipei, Taiwan; 9 Division of Cardiology, Heart Center, Cheng-Hsin General Hospital, and School of Medicine, National Yang-Ming University, Taipei, Taiwan; 10 Department of Medical Imaging and Radiological Sciences, I-Shou University and Division of Cardiology, Department of Internal Medicine, E-Da Hospital, Kaohsiung, Taiwan; 11 Cardiology Division of Cardiovascular Medical Center and Department of Nuclear Medicine, Far Eastern Memorial Hospital, New Taipei City, Taiwan; 12 School of Medicine, National Yang-Ming University, Taipei, Taiwan; 13 Division of Cardiology, Department of Internal Medicine, Kaohsiung Medical University Hospital and Kaohsiung Medical University, Kaohsiung, Taiwan; 14 Mackay Memorial Hospital, Mackay Medical College, New Taipei City, Taiwan; 15 Division of Cardiology, Department of Internal Medicine, China Medical University Hospital, Taichung, Taiwan; 16 Graduate Institute of Clinical Medical Science, China Medical University, Taichung, Taiwan; 17 Department of Cardiology, Buddhist Tzu-Chi General Hospital, Tzu-Chi University, Hualien, Taiwan; 18 Division of Cardiology, Department of Internal Medicine, National Taiwan University College of Medicine and Hospital, Taipei, Taiwan; 19 Department of Primary Care Medicine, College of Medicine, National Taiwan University, Taipei, Taiwan; 20 PhD Program in Translational Medicine, College of Medicine, Kaohsiung Medical University, Kaohsiung, Taiwan; 21 Department of Medical Research, Taipei Veterans General Hospital, Taipei, Taiwan

**Keywords:** Prognostic markers, Cardiovascular diseases

## Abstract

Polyunsaturated fatty acids (PUFAs) have been suggested for cardiovascular health. This study was conducted to investigate the prognostic impacts of the PUFA metabolites, oxylipins, on clinical outcomes in coronary artery disease (CAD). A total of 2,239 patients with stable CAD were prospectively enrolled and followed up regularly. Among them, twenty-five consecutive patients with new onset of acute myocardial infarction (AMI) within 2-year follow-up were studied. Another 50 gender- and age-matched patients without clinical cardiovascular events for more than 2 years were studied for control. Baseline levels of specific arachidonic acid metabolites were significantly higher in patients with subsequent AMI than in the controls. In Kaplan-Meier analysis, the incidence of future AMI was more frequently seen in patients with higher baseline levels of 8-hydroxyeicosatetraenoic acid (HETE), 9-HETE, 11-HETE, 12-HETE, 15-HETE, 19-HETE, 20-HETE, 5,6-epoxyeicosatrienoic acid (EET), 8,9-EET, 11,12-EET, or 14-15-EET when compared to their counterparts (all the *P* < 0.01). Further, serum levels of these specific HETEs, except for 11,12-EET, were positively correlated to the levels of some inflammatory and cardiac biomarker such as tumor necrosis factor-α and N-terminal pro B-type natriuretic peptide. Accordingly, serum specific oxylipins levels are increased and associated with the consequent onset of AMI, suggesting their potential role for secondary prevention in clinically stable CAD.

## Introduction

Coronary artery disease (CAD) is the leading cause of death worldwide^1^. Patients with established CAD are also at high risk of recurrent cardiovascular events^2^. However, the majority of ischemic events occur in people currently not identified by risk profiling^3^. Despite recent advances in pharmacological and invasive treatment, CAD remains a progressive disease even after successful percutaneous coronary intervention (PCI)^4,5^.

Polyunsaturated fatty acids (PUFAs) are thought beneficial to cardiovascular health. In cohort studies and randomized controlled trials, consuming PUFAs in place of saturated fatty acids (SFAs) or monounsaturated fatty acids (MUFAs) reduces risk of CAD^6–8^. A recent meta-analysis revealed that increasing PUFA intake could reduce risk of cardiovascular events^9^. PUFAs consist mainly of two families, omega-3 and omega-6. Omega-3 PUFAs including eicosapentaenoic acid (EPA) and docosahexaenoic acid (DHA) are usually found in fish oil. Omega-6 PUFAs including linoleic acid (LA) and arachidonic acid (AA) are mainly found in soybean oil. While PUFAs seem to be beneficial, there is still controversy about the role of omega-3 and/or omega-6 PUFA supplements for cardiovascular health^10–16^.

Oxylipins, a type of bioactive lipid mediators, are derived from the catalysis of PUFA substrates via lipoxygenases (LOXs), cyclooxygenases (COXs), or cytochrome P450s (CYPs)^17^. These metabolites exert a host of pathophysiological functions, such as cell proliferation^18,19^, inflammation^20^, inflammation resolution^21^, and vascular function^22^. It is well known that chronic vascular inflammation may contribute to the development and progression of atherosclerosis cardiovascular diseases including CAD as well as acute myocardial infarction (AMI). However, the potential roles of oxylipins in clinical cardiovascular diseases have not been well defined. Thus, this study aimed to investigate the prognostic impacts of serum oxylipins on clinical outcomes of CAD. In this study, ultra-performance liquid chromatography coupled with electrospray ionization tandem mass spectrometry (UPLC-ESI-MS/MS) was used for global profiling and analysis of the dynamics of oxylipins in patient sera. Baseline serum oxylipin profiles were determined to identify some specific oxylipins, if there were, that could be related to future development of AMI in a cohort of stable CAD patients. Our findings may provide a rationale to the potential roles of serum oxylipins, independent of PUFA, in clinical CAD.

## Materials and methods

### Study designs and study subjects

The current study is associated with the “*Development of New Biosignatures for Atherosclerosis Cardiovascular Diseases*” study, which is a multicenter study which enrolled a series of patients with stable CAD in 9 medical centers in Taiwan^23^. All of the patients were followed up regularly in each medical center. The study enrolled patients aged 20 years old or older, who had received successful PCI with either coronary stenting or balloon angioplasty or both at least once previously, and who had been in a stable condition on medical treatment for at least 1 month before enrollment. The study excluded patients who had been hospitalized for acute cardiovascular events within 3 months before enrollment, patients who were scheduled for further coronary revascularization or interventional procedures for specific cardiovascular diseases in the following year, patients who had malignancy or tumor diseases requiring either advanced medical or surgical therapy or both in the following year, patients who required hospitalization or operation for other major systemic diseases in the following year, or patients who were unable or unwilling to be followed up over the following year, or patients with life expectancy of less than 6 months or treatment with immunosuppressive agents. The subject inclusion criteria and exclusion criteria have be mentioned in previous study^23^.

In the current study, the study subjects were selected from the stable CAD patients who encountered new-onset of AMI within a 2-year follow-up period in the “*Development of New Biosignatures for Atherosclerosis Cardiovascular Diseases*” study. A gender- and age- matched control group was selected as a 2:1 fashion from those patients who did not encounter any cardiovascular events during a follow-up period for more than 2 years.

### Clinical data

The detailed medical history, personal history, family history, drug exposure history, and the use of food supplements of all the subjects were recorded. The demographic indexes including body weight, body height, blood pressure, waist and hip circumference were measured according to a standardized protocol by a well-trained nurse. Body mass index was defined as weight in kilograms divided by the square of height in meters. The waist-hip ratio was calculated as 100 × (waist circumference in centimeter/hip circumference in centimeter).

### Biomarker measurements

Fasting whole blood samples of the patients were obtained by venipuncture after 10-min rest in a supine position in the morning. The blood samples were centrifuged, and the sera were thawed for analysis. A series of biomarkers were checked, including high-sensitivity C-reactive protein (hs-CRP), adiponectin, lipoprotein-associated phospholipase A2 (Lp-PLA2), interleukin 6 (IL 6), tumor necrosis factor alpha (TNF-α), matrix metalloproteinase-9 (MMP-9), and N-terminal pro b-type natriuretic peptide (NT-pro BNP).

### Preparation of oxylipins

Patients’ sera (400 μL) were extracted with ice-cold CHCl_3_/MeOH (2:1, v/v) containing antioxidants 76 μM butylated hydroxytoluene (BHT, Acros Organics, USA) and 2.5 mM triphenylphosphine (TPP, Sigma-Aldrich, USA), and internal standards (Cayman Chemicals, USA) including 0.01 ppm each of 9-HODE-d4, PGE_2_-d4 and DHA-d5, and 0.1 ppm each of 20-HETE-d6, 5-HETE-d8, 14,15-EET-d11 and EPA-d5 in final methanol solution. All samples were kept at −20 °C for 30 min followed by centrifugation at 12,000 × g for 10 min at 4 °C. The organic layer was removed to a new Eppendorf tube, and the aqueous layer was extracted again with extraction solution. The organic layers of each sample were collected, concentrated, and redissolved in methanol for analysis by UPLC-ESI-MS/MS^24–27^.

### Quantitative profiling of oxylipins by UPLC-ESI-MS/MS

The system used for analysis was an UPLC system (Acquity UPLC, Waters, Millford, MA, USA) coupled with a TSQ Quantum Access Max (Thermo Fisher Scientific, San Jose, CA, USA) triple quadrupole mass spectrometer. The samples were separated using a BEH C18 column (particle size 1.7 μm, 2.1 × 100 mm, Waters, Milford, MA, USA) at 400 μL/min flow rate using 25 min gradient for analysis. A solvent gradient with mobile phase A contained 0.1% NH_4_OH in water and mobile phase B contained 0.1% NH_4_OH in MeOH was used for the separation. The solvent gradient was set as follows: 0–1 min, 92% A to B (isocratic); 1–15 min, 92–20% A to B (linear gradient); 15–18 min, 20% A to B (isocratic); 18–18.5 min, 20–0% A to B (linear gradient); 18.5–22 min, 100% B (isocratic); 22–22.5 min, 0–92% A to B (linear gradient); and 22.5–25 min, 92% A to B (isocratic). The instrument was operated in the negative multiple reaction-monitoring (MRM) mode. The conditions of MS operation were optimized as follows: vaporizer temperature, 300 °C; ion transfer capillary temperature, 270 °C; spray voltage, 2.7 kV; auxiliary gas (nitrogen), 10 Arb; sheath gas (nitrogen), 40 Arb; collision gas (argon) pressure, 1 mTorr. Quality control samples (QCs) were used to monitor the reproducibility of the separation system. Oxylipin standards dissolved in methanol were used for establishment of calibration curves for absolute quantification of oxylipins in samples. Chromatogram acquisition, detection of mass spectral peaks, and waveform processing were performed with ThermoXcalibur 2.1 SP1 software (Thermo Scientific, USA) and LCQuan 2.6.1 software (Thermo Scientific, USA). The peak area of each quantified ion was calculated and then normalized against the peak area of the corresponding internal standards^24,25^. The optimized MS/MS conditions and limits of quantification (LOQ) for each of the oxylipins were presented in Supplemental Table 1.

### Reagents

5,6-EET and 14,15-EET were purchased from Cayman Chemical (Ann Arbor, MI, USA).

### Endothelial cell culture

Human coronary artery endothelial cells (HCAECs) were isolated from the coronary artery of donors and purchased from ScienCell Research Laboratories (Carlsbad, CA, USA). HCAECs were cultured on fibronectin-coated plates with endothelial cell medium containing 5% fetal bovine serum, 1% penicillin/streptomycin solution and 1% endothelial cell growth supplement. In this experiment, HCAECs with 2 to 6 passages were used. Cells were grown in a 5% CO_2_ humidified atmosphere at 37 °C. Fresh medium was replenished every 3 days.

### THP-1 cell culture

THP-1 cells were purchased from ATCC (Manassas, VA, USA), and incubated at 37 °C in 5% CO_2_. THP-1 cells were cultured in RPMI 1640 medium (Gibco) supplemented with 10% fetal bovine serum (Gibco).

### THP-1 Cell and endothelial cell adhesion assay

Confluent HCAECs on plates were incubated with fluorescent probe labelled THP-1 cells (5 × 10^5^ cells/mL) at 37 °C for 1 h. After gently washing with PBS, non-adherent THP-1 cells were removed, and the adhered THP-1 cells in four fields per 200× high-power field well, and in six randomly chosen high-power fields per well were counted using a fluorescence microscope (Zeiss, Axiovert 200 M).

### Data analysis

Statistical analysis was carried out by using SPSS software (Version 18.0, SPSS, Chicago, Illinois, USA). Data were presented as frequency (percentage) or mean ± standard deviation. Continuous parametric data between two patient groups were compared by using unpaired Student’s *t*-test, and nonparametric data by the Mann-Whitney test. Categorical data between two patient groups was compared by Chi-square test with Yates’ correction or Fisher’s exact test, whichever was appropriate. The relative levels of oxylipins and biomarkers were evaluated by using Pearson’s correlation coefficient. Multivariate data analysis was carried out with SIMCA-P 11.0 software (Umetrics, Umeå, Sweden). Clustering and analysis of the metabolic alterations in groups was performed by using partial least squares discriminant analysis (PLS-DA). CIMminer (heat map) was used to reveal the fold change of each metabolite. Receiver operating characteristic (ROC) curve analysis was performed for the optimal cut-off value of oxylipins to differentiate stable CAD patients with future AMI within 2 years from those without any events within 2 years. Survival analysis was conducted by using Kaplan-Meier analysis, with significance determined by the log-rank test. Cox proportional hazard regression models were used to assess the association between oxylipins and future AMI. Both crude hazard ratios (HRs) and adjusted HRs were determined after adjusting for potential confounding factors. HRs of oxylipins for future AMI were adjusted for gender, age, waist-hip ratio, and body mass index. Two-sided *P* values less than 0.05 were considered statistically significant.

### Ethics statement

The study was conducted in accordance with the Declaration of Helsinki. It was approved by the independent review boards (IRBs) and independent ethics committees of the following hospitals: Taipei Veterans General Hospital, E-Da Hospital, Far Eastern Memorial Hospital, Cheng-Hsin General Hospital, Kaohsiung Medical University Hospital, Mackay Memorial Hospital, China Medical University Hospital, Buddhist Tzu-Chi General Hospital, and National Taiwan University Hospital. The study was also approved by the Joint IRB Ethics Committees Review Boards in Taiwan. All patients agreed to participate the study and gave the study’s informed consent form before they entered the study.

## Results

### Demographic data of patients

**Figure 1:**
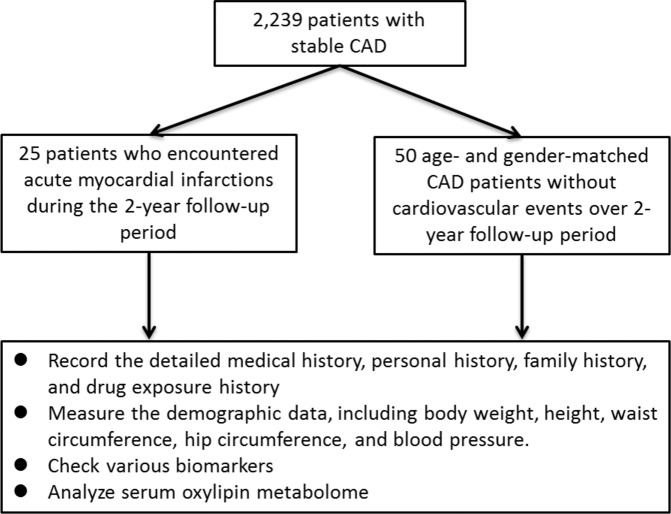
Flow chart of the study.

A total of 2,239 patients with stable CAD who had undergone PCI were enrolled and followed up regularly in the “*Development of New Biosignatures for Atherosclerosis Cardiovascular Diseases*” study. Among them, the 25 consecutive patients encountering new-onset of AMI during the 2-year follow-up period were studied as the subject group. Another 50 gender- and age-matched CAD patients without any cardiovascular events over the follow-up period for more than 2 years were selected as the control group (Fig. 1). The baseline characteristics, including age, gender, waist-hip ratio, body mass index, hypertension, diabetes, smoking, drinking, and concomitant medications were similar in the 2 groups (Table 1). Further, the dietary records from the patients are organized in Supplemental Table 2. Only 6% of control group took fish oil, and no one in the subject group. There were no special foods/supplements taken by the patients in the two groups which could contribute to affecting PUFAs levels in patient’s sera; in other words, the demographic data are similar in the two groups. To make sure that all the control patients may not suffer from any new-onset cardiovascular events including AMI in a sufficient period, the follow-up duration was significantly longer in control patients than in study subjects (control group vs. subject group = 45 ± 5 months vs. 11 ± 11 months, *P* < 0.001).

**Table 1:** Baseline characteristics and inflammatory biomarker profiles of the patients with stable coronary artery disease who encountered acute myocardial infarction during follow-up (Subject group) and those patients without cardiovascular events during follow-up (Control group).

	Control group (*n* = 50)	Subject group (*n* = 25)	*P* value
Age, years	64.4 ± 9.9	63.2 ± 14.9	0.694
Male, n (%)	46 (92.0%)	23 (92.0%)	1.000
Waist circumference, cm	93.4 ± 8.6	93.8 ± 7.4	0.807
Hip circumference, cm	97.8 ± 6.5	100.1 ± 5.3	0.102
Waist-hip ratio	1.0 ± 0.1	0.9 ± 0.1	0.223
Height, cm	165.2 ± 5.6	167.1 ± 7.7	0.292
Body weight, kgw	70.0 ± 10.0	72.8 ± 11.8	0.326
BMI	25.6 ± 3.4	26.0 ± 3.3	0.662
SBP, mmHg	132.1 ± 17.6	135.2 ± 15.0	0.439
DBP, mmHg	76.4 ± 11.2	80.4 ± 15.3	0.255
Hypertension, n (%)	25 (50.0%)	12 (48.0%)	0.870
Diabetes mellitus, n (%)	27 (54.0%)	12 (48.0%)	0.624
History of smoking, n (%)	25 (50.0%)	15 (60.0%)	0.413
History of drinking, n (%)	11 (22.0%)	2 (8.0%)	0.198
Anticoagulants, n (%)	0 (0.0%)	1 (4.0%)	0.333
Antiplatelet, n (%)	48 (96.0%)	23 (92.0%)	0.597
ACEI/ARB, n (%)	30 (60.0%)	15 (60.0%)	1.000
B-blocker, n (%)	27 (54.0%)	14 (56.0%)	0.870
CCB, n (%)	27 (54.0%)	10 (40.0%)	0.253
Diuretics, n (%)	6 (12.0%)	4 (16.0%)	0.723
Statins, n (%)	30 (60.0%)	18 (72.0%)	0.307
Total cholesterol, mg/dL	165.8 ± 31.6	157.3 ± 29.5	0.257
Triglyceride, mg/dL	124.8 ± 67.6	128.7 ± 75.3	0.829
HDLC, mg/dL	42.5 ± 11.7	38.7 ± 10.6	0.173
LDLC, mg/dL	96.3 ± 29.7	93.1 ± 24.3	0.625
hs-CRP, mg/dL	0.2 ± 0.3	0.3 ± 0.2	0.684
Adiponectin, ng/mL	18.3 ± 34.0	14.1 ± 10.4	0.421
Lp-PLA2, ng/mL	80.3 ± 121.0	128.2 ± 189.8	0.295
IL 6, pg/mL	2.6 ± 2.9	2.7 ± 2.7	0.891
TNF-α, pg/mL	4.6 ± 4.7	5.1 ± 5.5	0.738
MMP-9, ng/mL	472.2 ± 291.0	416.1 ± 358.5	0.530
NT-pro BNP, pg/mL	366.8 ± 555.9	521.5 ± 734.9	0.359

ACEI, angiotensin converting enzyme inhibitor; ARB, angiotensin II receptor blocker; BMI, body mass index; CCB, calcium channel blocker; DBP, diastolic blood pressure; hs-CRP, high-sensitivity C-reactive protein; IL 6, interleukin 6; Lp-PLA2, lipoprotein-associated phospholipase A2; MMP-9, matrix metalloproteinase-9; NT-pro BNP, N-terminal pro b-type natriuretic peptide; SBP, systolic blood pressure; TNF-α, tumor necrosis factor alpha.

### Circulating oxylipin metabolome profiles of the patients

**Figure 2:**
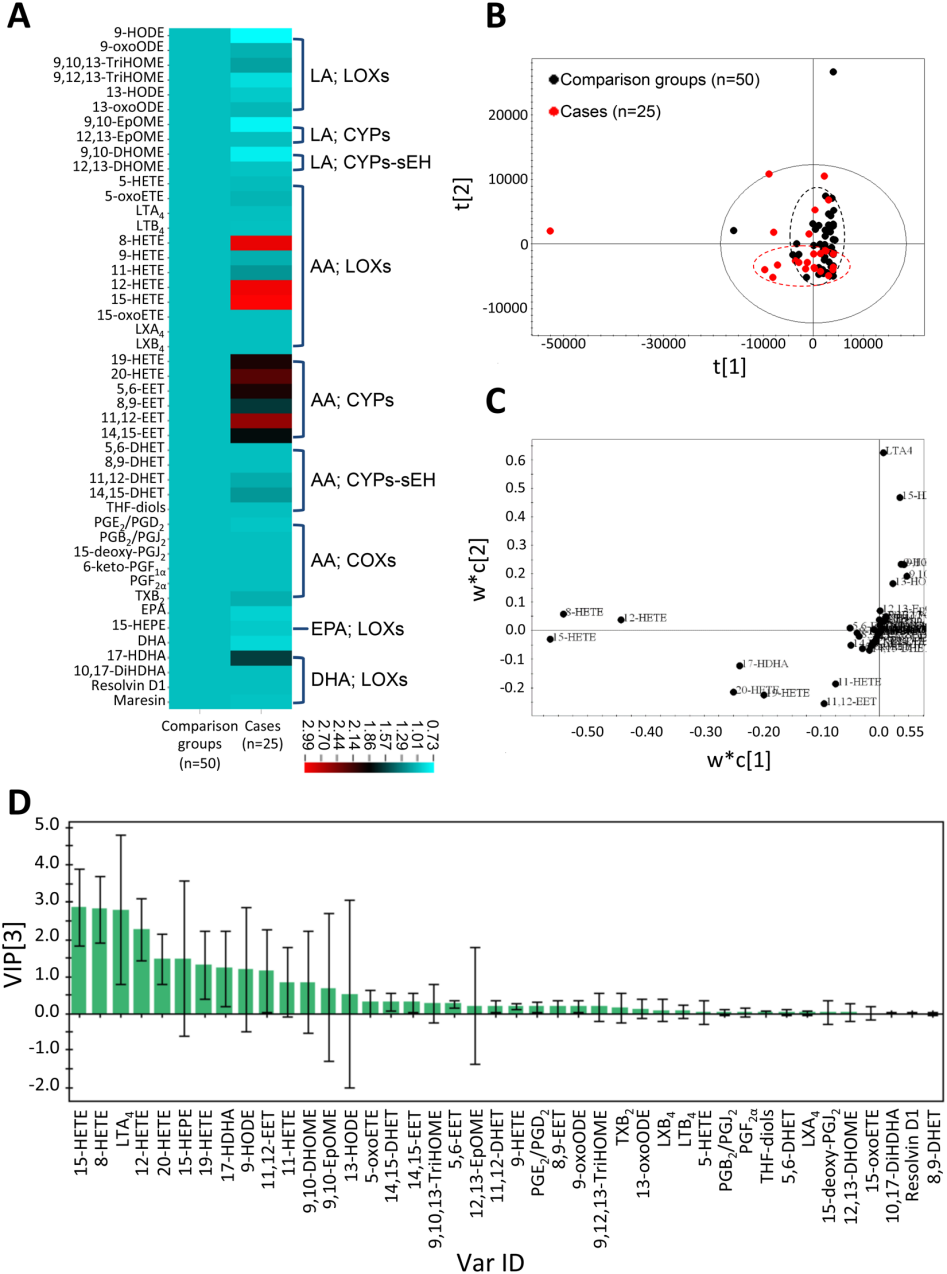
Comparative oxylipin metabolite analysis of sera from CAD patients (subject group, *n* = 25; control group, *n* = 50). (**A**) The corresponding heat map highlights the bioactive lipid metabolites classified according to precursors and the corresponding metabolic enzymes. The fold-change of specific metabolite of subject vs. control groups are presented. (**B**) Score plot generated from PLS-DA show distinct clustering between groups. (**C**) Loading plot generated from PLS-DA show distinct clustering between groups. **(D**) VIP reflects the most important variables (oxylipin species) over the model as a whole.

In order to compare the differences between 2 patient groups, an UPLC-MS/MS-based targeted oxylipin metabolomics platform in a MRM model developed in-house covering 46 AA, LA, EPA and DHA oxidized fatty acid metabolites was used to investigate the oxylipin dynamics in the serum samples from the study cohort of study subjects (subject group, *n* = 25) and control patients (control group, *n* = 50). Identity and absolute quantification of 46 oxylipin metabolites (ng/mL), which were detected with highly reproducible signals are presented in Table 2. Heat map-generated color-coded images represent the fold-change value calculated from quantified data of oxylipins. The data in Fig. 2A reveal that two types of oxylipins, *i.e*., HETEs and EETs derived from AAs which were obviously increased in the subject group compared to the control group. The analytical method PLS-DA was employed to compare the metabolite distribution between groups. The score plot shows that the oxylipin distribution from patients in the subject group (red dots) and control group (black dots) can be distinguished (Fig. 2B). The loading plot and VIP suggest that a few of the oxylipin metabolites, such as 8-hydroxyeicosatetraenoic acid (8-HETE), 12-HETE, and 15-HETE are distinct in this oxylipin metabolome analysis (Fig. 2C,D).

**Table 2:** Baseline serum oxylipin metabolite profiles in patients with stable coronary artery disease who encountered acute myocardial infarction during follow-up (Subject group) and in the patients without cardiovascular events during follow-up (Control group).

Substrate	Catalytic enzyme	Oxylipin (ng/mL)	Control group (*n* = 50)	Subject group (*n* = 25)	*P* value
LA; ω-6	LOXs	9-HODE	14.53 ± 18.29	10.53 ± 6.41	0.216
		9-oxoODE	0.65 ± 0.08	0.69 ± 0.13	0.164
		9,10,13-TriHOME	1.14 ± 0.34	1.29 ± 0.60	0.257
		9,12,13-TriHOME	1.20 ± 1.41	1.05 ± 0.43	0.480
		13-HODE	21.01 ± 17.33	20.16 ± 12.26	0.813
		13-oxoODE	0.72 ± 0.16	0.75 ± 0.16	0.439
	CYPs	9,10-EpOME	14.73 ± 14.21	11.14 ± 6.27	0.169
		12,13-EpOME	20.40 ± 16.15	20.36 ± 11.40	0.991
	CYPs-sEH	9,10-DHOME	7.17 ± 8.56	5.66 ± 5.15	0.351
		12,13-DHOME	1.48 ± 1.06	1.45 ± 0.86	0.893
AA; ω-6	LOXs	5-HETE	4.55 ± 0.62	4.63 ± 0.48	0.529
		5-oxoETE	4.48 ± 0.55	4.72 ± 0.51	0.069
		LTA_4_	280.25 ± 243.41	279.20 ± 235.88	0.986
		LTB_4_	4.21 ± 0.37	4.16 ± 0.04	0.361
		8-HETE	30.48 ± 42.97	88.35 ± 142.48	0.011
		9-HETE	4.24 ± 0.19	4.52 ± 0.67	0.007
		11-HETE	4.51 ± 0.38	5.38 ± 1.66	0.001
		12-HETE	19.08 ± 25.39	55.99 ± 87.64	0.009
		15-HETE	30.63 ± 42.31	91.48 ± 145.66	0.009
		15-oxoETE	4.16 ± 0.04	4.16 ± 0.05	0.860
		LXA_4_	4.13 ± 0.00	4.13 ± 0.01	0.338
		LXB_4_	4.47 ± 0.21	4.48 ± 0.27	0.942
	CYPs	19-HETE	6.41 ± 3.19	12.64 ± 12.29	0.001
		20-HETE	8.44 ± 5.93	18.95 ± 21.70	0.003
		5,6-EET	40.26 ± 31.06	79.12 ± 99.03	0.015
		8,9-EET	26.71 ± 13.14	43.10 ± 34.93	0.005
		11,12-EET	68.37 ± 43.73	171.77 ± 203.83	0.001
		14,15-EET	38.68 ± 17.78	69.53 ± 67.05	0.004
	CYPs-sEH	5,6-DHET	20.76 ± 0.49	20.85 ± 0.49	0.456
		8,9-DHET	20.97 ± 0.51	20.99 ± 0.60	0.894
		11,12-DHET	27.23 ± 6.06	29.76 ± 9.31	0.169
		14,15-DHET	36.80 ± 9.55	43.37 ± 23.12	0.094
		THF-diols	20.71 ± 0.28	20.63 ± 0.13	0.148
	COXs	PGE_2_/PGD_2_	0.07 ± 0.01	0.07 ± 0.00	0.152
		PGB_2_/PGJ_2_	0.07 ± 0.00	0.07 ± 0.00	0.837
		15-deoxy-PGJ_2_	0.09 ± 0.03	0.09 ± 0.02	0.937
		6-keto-PGF_1α_	0.07 ± 0.00	0.07 ± 0.00	0.931
		PGF_2α_	0.07 ± 0.00	0.07 ± 0.00	0.704
		TXB_2_	0.07 ± 0.02	0.08 ± 0.03	0.475
EPA; ω-3			114.86 ± 100.70	106.17 ± 94.60	0.716
	LOXs	15-HEPE	161.12 ± 69.18	155.45 ± 72.28	0.747
DHA; ω-3			2341.65 ± 1424.61	2074.06 ± 1612.37	0.496
	LOXs	17-HDHA	137.19 ± 135.15	216.64 ± 231.91	0.085
		10,17-DiHDHA	5.51 ± 0.03	5.52 ± 0.03	0.157
		Resolvin D1	5.50 ± 0.01	5.51 ± 0.03	0.203
		Maresin	5.60 ± 0.21	5.49 ± 0.01	0.252

AA, arachidonic acid; COX, cyclooxygenase; CYP, cytochrome P450; CYP-sEH, cytochrome P450-soluble epoxide hydrolase; DHA, docosahexaenoic acid; EPA, eicosapentaenoic acid; LA, linoleic acid; LOX, lipoxygenase; sEH, soluble epoxide hydrolase.

Overall, serum levels of LA and AA were similar in the 2 groups (LA: control group vs. subject group = 58060.2 ± 13094.1 ng/mL vs. 63826.8 ± 20874.8 ng/mL, *P* = 0.594; AA: control group vs. subject group = 3828.5 ± 1132.9 ng/mL vs. 5469.4 ± 1842.8 ng/mL, *P* = 0.121). In comparison with the control groups, patients with subsequent AMI (subject group) had higher baseline levels of specific LOX catalyzed AA metabolites, including 8-HETE (control group vs. subject group = 30.48 ± 42.97 ng/mL vs. 88.35 ± 142.48 ng/mL, *P* = 0.011), 9-HETE (control group vs. subject group = 4.24 ± 0.19 ng/mL vs. 4.52 ± 0.67 ng/mL, *P* = 0.007), 11-HETE (control group vs. subject group = 4.51 ± 0.38 ng/mL vs. 5.38 ± 1.66 ng/mL, *P* = 0.001), 12-HETE (control group vs. subject group = 19.08 ± 25.39 ng/mL vs. 55.99 ± 87.64 ng/mL, *P* = 0.009), and 15-HETE (control group vs. subject group = 30.63 ± 42.31 ng/mL vs. 91.48 ± 145.66 ng/mL, *P* = 0.009) (Table 2).

Further, patients who experienced subsequent AMI (study group) had higher baseline levels of specific CYP catalyzed AA metabolites, including 19-HETE (control group vs. subject group = 6.41 ± 3.19 ng/mL vs. 12.64 ± 12.29 ng/mL, *P* = 0.001), 20-HETE (control group vs. subject group = 8.44 ± 5.93 ng/mL vs. 18.95 ± 21.70 ng/mL, *P* = 0.003), 5,6-epoxyeicosatrienoic acid (5,6-EET) (control group vs. subject group = 40.26 ± 31.06 ng/mL vs. 79.12 ± 99.03 ng/mL, *P* = 0.015), 8,9-EET (control group vs. subject group = 26.71 ± 13.14 ng/mL vs. 43.10 ± 34.93 ng/mL, *P* = 0.005), 11,12-EET (control group vs. subject group = 68.37 ± 43.73 ng/mL vs. 171.77 ± 203.83 ng/mL, *P* = 0.001), and 14,15-EET (control group vs. subject group = 38.68 ± 17.78 ng/mL vs. 69.53 ± 67.05 ng/mL, *P* = 0.004) (Table 2). The baseline levels of other oxylipins, including LA metabolites, EPA metabolites, and DHA metabolites were similar in the control group and in the subject group (Table 2).

### Correlation of oxylipins and selected inflammatory and cardiac biomarkers

In order to clarify the potential roles of these identified oxilipins, we compared the baseline levels of 11 oxylipins that were present in the subject group patients at a significantly higher level relative to the control group patients, with the baseline levels of 7 biomarkers, hs-CRP, adiponectin, Lp-PLA2, IL 6, TNF-α, MMP-9, and NT-pro-BNP. It was shown that TNF-α levels were correlated to 10 AA metabolites, including 8-HETE (r = 0.294, *P* = 0.015), 9-HETE (r = 0.316, *P* = 0.009), 11-HETE (r = 0.271, *P* = 0.025), 12-HETE (r = 0.292, *P* = 0.016), 15-HETE (r = 0.293, *P* = 0.015), 19-HETE (r = 0.298, *P* = 0.014), 20-HETE (r = 0.302, *P* = 0.013), 5,6-EET (r = 0.302, *P* = 0.012), 8,9-EET (r = 0.340, *P* = 0.004), and 14,15-EET (r = 0.252, *P* = 0.037). Besides, the NT-pro-BNP levels were correlated to 10 AA metabolites, including 8-HETE (r = 0.438, *P* < 0.001), 9-HETE (r = 0.425, *P* < 0.001), 11-HETE (r = 0.233, *P* = 0.048), 12-HETE (r = 0.437, *P* < 0.001), 15-HETE (r = 0.437, *P* < 0.001), 19-HETE (r = 0.413, *P* < 0.001), 20-HETE (r = 0.433, *P* < 0.001), 5,6-EET (r = 0.427, *P* < 0.001), 8,9-EET (r = 0.393, *P* = 0.001), and 14,15-EET (r = 0.394, *P* = 0.001). There were no correlations of the baseline levels of the 11 oxylipins with the baseline levels of hs-CRP, adiponectin, Lp-PLA2, IL 6, and MMP-9 (Table 3).

**Table 3:** Correlation of serum levels of the 11 arachidonic acid metabolites with that of the inflammatory biomarkers in 75 patients with stable coronary artery disease.

		hs-CRP	Adiponectin	LppPLA_2_	IL6	TNF-α	MMP-9	NT-pro-BNP
8-HETE	r	−0.042	−0.010	0.186	0.010	0.294	0.105	0.438
	*P* value	0.737	0.936	0.128	0.934	<0.05	0.395	<0.001
9-HETE	r	−0.028	−0.011	0.203	0.005	0.316	0.104	0.425
	*P* value	0.819	0.931	0.098	0.970	<0.01	0.397	<0.001
11-HETE	r	0.063	−0.016	0.196	0.120	0.271	0.108	0.233
	*P* value	0.608	0.898	0.110	0.331	<0.05	0.382	0.048
12-HETE	r	−0.043	−0.011	0.192	0.001	0.292	0.102	0.437
	*P* value	0.729	0.931	0.120	0.991	<0.05	0.412	<0.001
15-HETE	r	−0.037	−0.013	0.187	0.007	0.293	0.106	0.437
	*P* value	0.762	0.919	0.126	0.952	<0.05	0.391	<0.001
19-HETE	r	−0.008	−0.004	0.216	0.005	0.298	0.100	0.413
	*P* value	0.949	0.973	0.077	0.970	<0.05	0.417	<0.001
20-HETE	r	−0.025	−0.010	0.204	0.018	0.302	0.104	0.433
	*P* value	0.840	0.934	0.098	0.888	<0.05	0.401	<0.001
5,6-EET	r	−0.035	−0.012	0.176	0.003	0.302	0.090	0.427
	*P* value	0.774	0.923	0.148	0.978	<0.05	0.463	<0.001
8,9-EET	r	−0.042	−0.014	0.190	−0.006	0.340	0.128	0.393
	*P* value	0.732	0.909	0.117	0.963	<0.005	0.295	0.001
11,12-EET	r	0.053	−0.046	0.200	0.115	0.214	0.092	0.168
	*P* value	0.667	0.708	0.100	0.346	0.077	0.450	0.157
14,15-EET	r	−0.019	−0.008	0.163	0.008	0.252	0.080	0.394
	*P* value	0.874	0.951	0.180	0.945	<0.05	0.516	0.001

Pearson’s correlation coefficient was used to evaluate the relation between the levels of arachidonic acid metabolites and biomarkers. hs-CRP, high-sensitivity C-reactive protein; IL 6, interleukin 6; Lp-PLA2, lipoprotein-associated phospholipase A2; MMP-9, matrix metalloproteinase-9; NT-pro BNP, N-terminal pro b-type natriuretic peptide; TNF-α, tumor necrosis factor-alpha.

### Oxylipins as predictors of future AMI

ROC curve analysis was conducted to identify the optimal cut-off value of 11 AA metabolites to differentiate stable CAD patients with future AMI within 2 years from those without any events within 2 years. The optimal cut-off values were 30.92 ng/mL for 8-HETE, 4.28 ng/mL for 9-HETE, 4.77 ng/mL for 11-HETE, 17.39 ng/mL for 12-HETE, 32.37 ng/mL for 15-HETE, 6.03 ng/mL for 19-HETE, 11.34 ng/mL for 20-HETE, 34.42 ng/mL for 5,6-EET, 24.50 ng/mL for 8,9-EET, 74.26 ng/mL for 11,12-EET, and 44.53 ng/mL for 14,15-EET (Supplemental Table 3).

**Figure 3:**
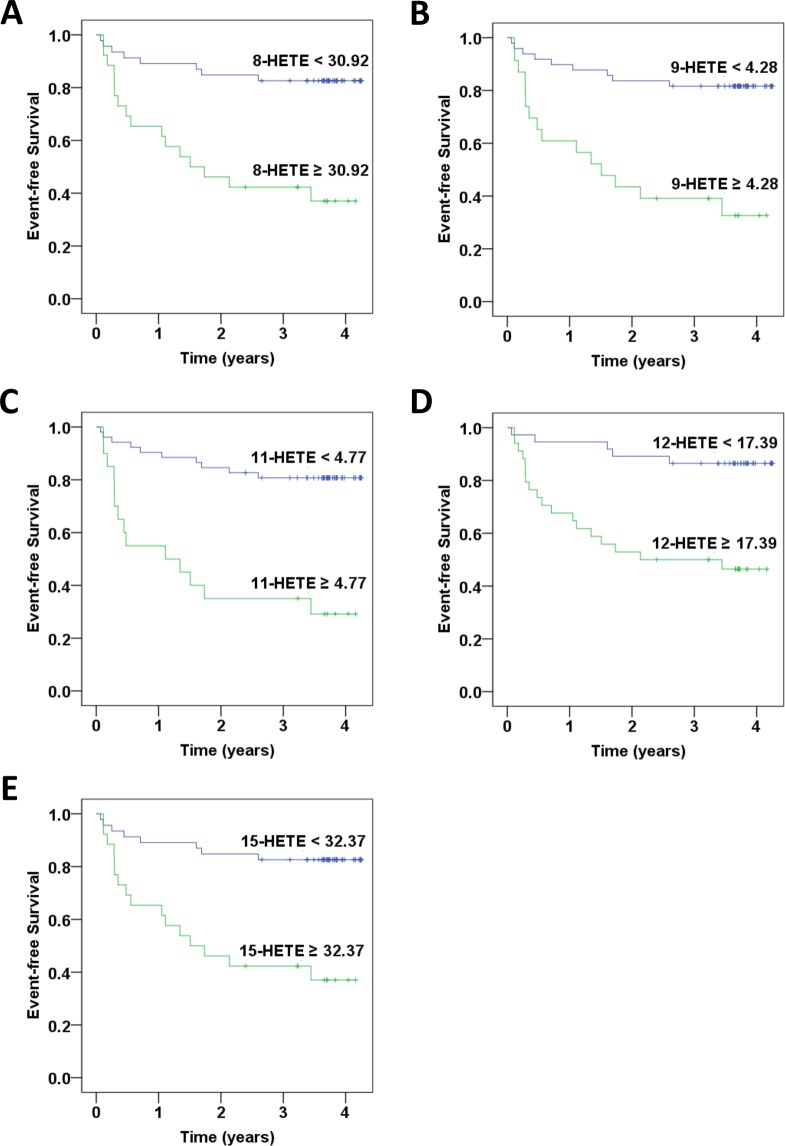
Kaplan–Meier curves of outcomes associated with specific lipoxygenases (LOXs) catalyzed arachidonic acid metabolites in patients with coronary artery disease. The rates of freedom from acute myocardial infarction are shown: (**A**) 8-HETE ≥ 30.92 ng/mL vs. 8-HETE < 30.92 ng/mL; (**B)** 9-HETE ≥ 4.28 ng/mL vs. 9-HETE < 4.28 ng/mL (p < 0.001); (**C**) 11-HETE ≥ 4.77 ng/mL vs. 11-HETE < 4.77 ng/mL; (**D**) 12-HETE ≥ 17.39 ng/mL vs. 12-HETE < 17.39 ng/mL; (**E**) 15-HETE ≥ 32.37 ng/mL vs. 15-HETE < 32.37 ng/mL.

**Figure 4:**
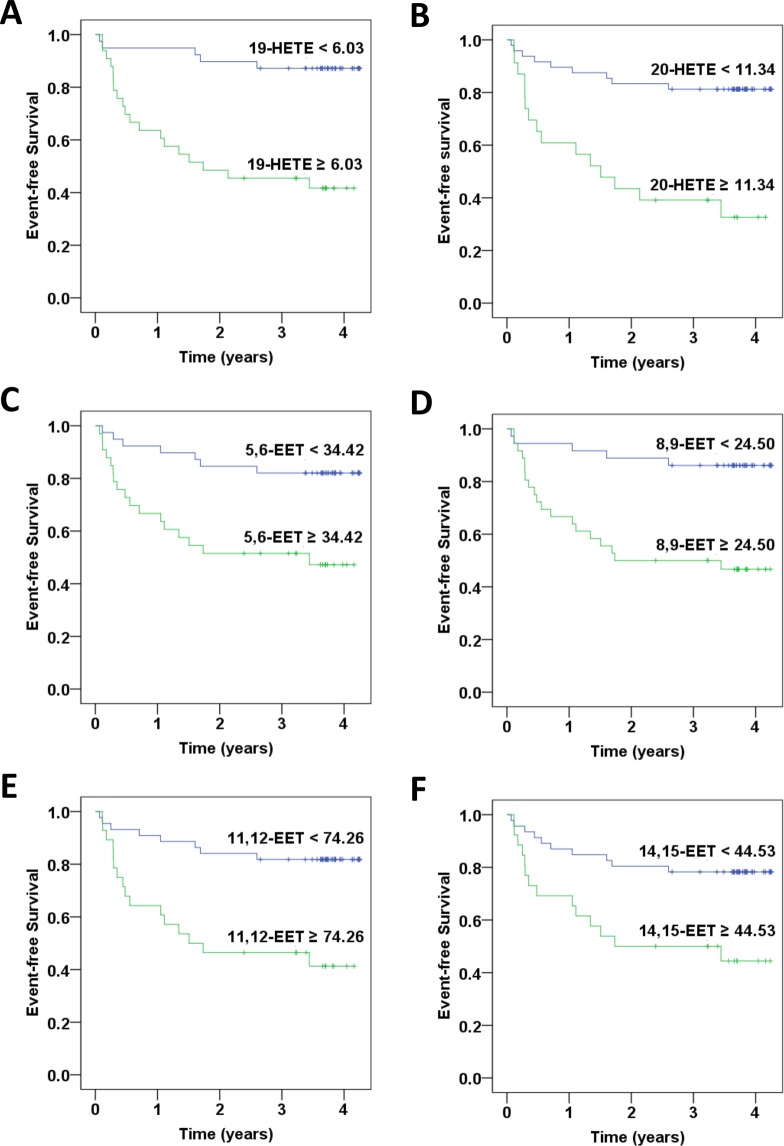
Kaplan–Meier curves of outcomes associated with specific cytochrome P450s (CYPs) catalyzed arachidonic acid metabolites in patients with coronary artery disease. The rates of freedom from acute myocardial infarction are shown: (**A**) 19-HETE ≥ 6.03 ng/mL vs.19-HETE < 6.03 ng/mL; (**B**) 20-HETE ≥ 11.34 ng/mL vs. 20-HETE < 11.34 ng/mL; (**C**) 5,6-EET ≥ 34.42 ng/mL vs. 5,6-EET < 34.42 ng/mL; (**D**) 8,9-EET ≥ 24.50 ng/mL vs. 8,9-EET < 24.50 ng/mL; **(E**) 11,12-EET ≥ 74.26 ng/mL vs. 11,12-EET < 74.26 ng/mL; (**F**) 14,15-EET ≥ 44.53 ng/mL vs. 14,15-EET < 44.53 ng/mL.

According to a Kaplan-Meier analysis, the incidence of new-onset AMI was significantly higher in the stable CAD patients with baseline 8-HETE ≥ 30.92 ng/mL (*P* < 0.001), 9-HETE ≥ 4.28 ng/mL (*P* < 0.001), 11-HETE ≥ 4.77 ng/mL (*P* < 0.001), 12-HETE ≥ 17.39 ng/mL (*P* < 0.001), 15-HETE ≥ 32.37 ng/mL (*P* < 0.001), 19-HETE ≥ 6.03 ng/mL (*P* < 0.001), 20-HETE ≥ 11.34 ng/mL (*P* < 0.001), 5,6-EET ≥ 34.42 ng/mL (*P* = 0.002), 8,9-EET ≥ 24.50 ng/mL (*P* < 0.001), 11,12-EET ≥ 74.26 ng/mL (*P* < 0.001), or 14–15-EET ≥ 44.53 ng/mL (*P* = 0.004) when compared to their counterparts (Figs. 3 and 4). These findings suggest that higher specific oxylipins were related to new-onset AMI.

In order to eliminate the possible impact of confounding factors, multivariate Cox regression analysis was performed. The results consistently showed that patients with baseline 8-HETE ≥ 30.92 ng/mL (HR, 5.11; 95% CI, 2.13–12.25), *P* < 0.001), 9-HETE ≥ 4.28 ng/mL (HR, 5.19; 95% CI, 2.21–12.22), *P* < 0.001), 11-HETE ≥ 4.77 ng/mL (HR, 5.99; 95% CI, 2.55–14.10), *P* < 0.001), 12-HETE ≥ 17.39 ng/mL (HR, 5.38; 95% CI, 1.97–14.66), *P* = 0.001), 15-HETE ≥ 32.37 ng/mL (HR, 5.11; 95% CI, 2.13–12.25), *P* < 0.001), 19-HETE ≥ 6.03 ng/mL (HR, 7.39; 95% CI, 2.65–20.60), *P* < 0.001), 20-HETE ≥ 11.34 ng/mL (HR, 5.33; 95% CI, 2.25–12.66), *P* < 0.001), 5,6-EET ≥ 34.42 ng/mL (HR, 3.78; 95% CI, 1.53–9.36), *P* = 0.004), 8,9-EET ≥ 24.50 ng/mL (HR, 5.11; 95% CI, 1.87–14.02), *P* = 0.002), 11,12-EET ≥ 74.26 ng/mL (HR, 4.43; 95% CI, 1.86–10.55), *P* = 0.001), or 14,15-EET ≥ 44.53 ng/mL (HR, 3.11; 95% CI, 1.35–7.14), *P* = 0.007) had significantly increased risk of future AMI compared to their counterparts (Table 4).

**Table 4:** Univariate and multivariate analysis of baseline oxylipins levels as the predictors of subsequent acute myocardial infarction in 75 patients with stable coronary artery disease.

Oxylipins	Univariate analysis		Multivariate analysis	
	HR (95% CI)	*P* value	HR (95% CI)	*P* value
8-HETE ≥ 30.92 ng/mL	4.82(2.05–11.35)	<0.001	5.11(2.13–12.25)	<0.001
9-HETE ≥ 4.28 ng/mL	5.02(2.18–11.56)	<0.001	5.19(2.21–12.22)	<0.001
11-HETE ≥ 4.77 ng/mL	5.55(2.44–12.60)	<0.001	5.99(2.55–14.10)	<0.001
12-HETE ≥ 17.39 ng/mL	5.27(1.95–14.24)	0.001	5.38(1.97–14.66)	0.001
15-HETE ≥ 32.37 ng/mL	4.82(2.05–11.35)	<0.001	5.11(2.13–12.25)	<0.001
19-HETE ≥ 6.03 ng/mL	6.23(2.32–16.79)	<0.001	7.39(2.65–20.60)	<0.001
20-HETE ≥ 11.34 ng/mL	4.91(2.13–11.30)	<0.001	5.33(2.25–12.66)	<0.001
5,6-EET ≥ 34.42 ng/mL	3.77(1.56–9.12)	0.003	3.78(1.53–9.36)	0.004
8,9-EET ≥ 24.50 ng/mL	4.94(1.84–13.27)	0.002	5.11(1.87–14.02)	0.002
11,12-EET ≥ 74.26 ng/mL	4.21(1.79–9.89)	0.001	4.43(1.86–10.55)	0.001
14–15-EET ≥ 44.53 ng/mL	3.14(1.39–7.10)	0.006	3.11(1.35–7.14)	0.007

Multivariate analysis was conducted by adjusting age, gender, waist-hip ratio, and body mass index.

CI, confidence interval; HR, hazard ratio.

### Pretreatment with 5,6-EET or 14,15-EET increased the adhesiveness of HCAECs to THP-1 Cells

**Figure 5:**
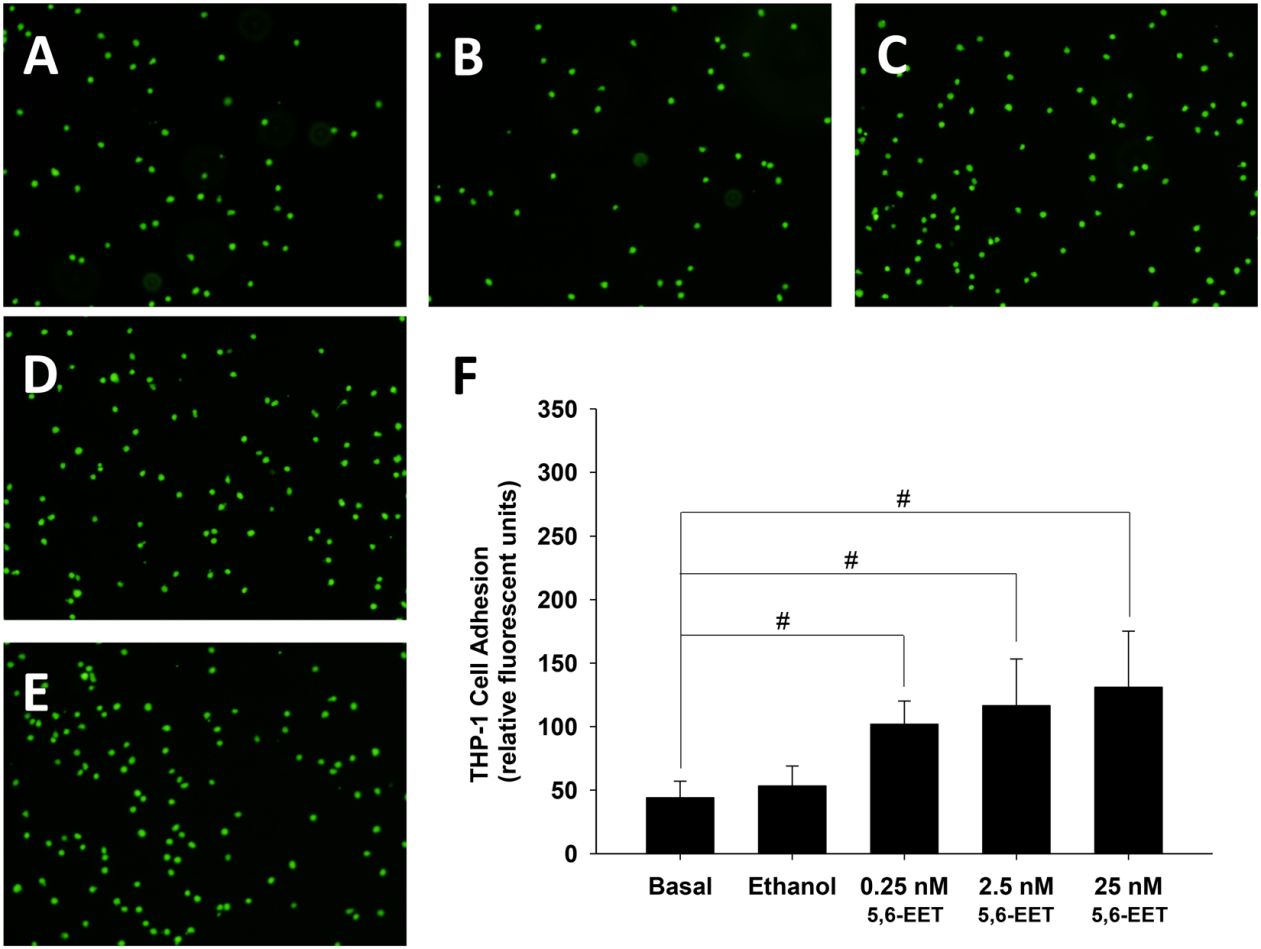
Pretreatment with 5,6-EET increased the adhesiveness of HCAECs to THP-1 cells. HCAECs were untreated (**A**) or treated with vehicle (**B**), 0.25 nM (**C**), 2.5 nM **(D**), or 25 nM (**E**) 5,6-EET for 24 h. The amount of THP-1 cell adherence is represented as relative fluorescence units. Pretreatment with 0.25 nM, 2.5 nM, or 25 nM 5,6-EET significantly and dose-dependently increased the adhesiveness of HCAECs to THP-1 cells (*P* < 0.001) (**F**).

**Figure 6:**
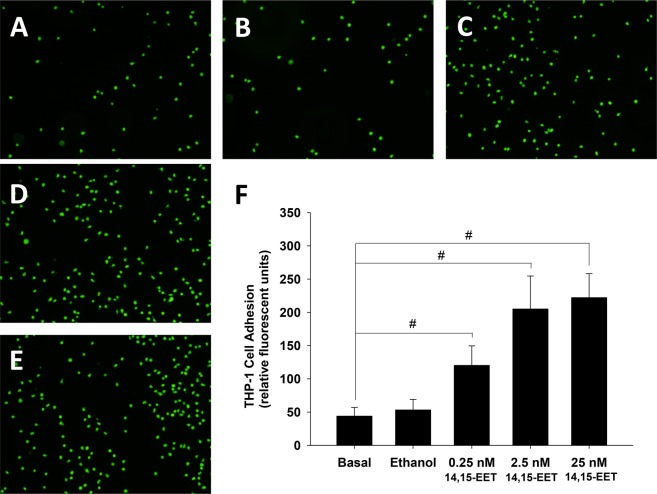
Pretreatment with 14,15-EET increased the adhesiveness of HCAECs to THP-1 cells. HCAECs were untreated (**A**) or treated with vehicle (**B**), 0.25 nM (**C**), 2.5 nM (**D**), or 25 nM (**E**) 14,15-EET for 24 h. The amount of THP-1 cell adherence is represented as relative fluorescence units. Pretreatment with 0.25 nM, 2.5 nM, or 25 nM 5,6-EET significantly and dose-dependently increased the adhesiveness of HCAECs to THP-1 cells (*P* < 0.001) (**F**).

HCAECs were pretreated with 0.25 nM, 2.5 nM, and 25 nM of 5,6-EET or 14,15-EET for 24 h, then co-cultured with BCECF-AM–labeled THP-1 cells. The THP-1 cells adherence/binding to HCAECs was quantified. The data in Figs. 5 and 6 show the increase of adhered fluorescent THP-1 cells when they were co-cultured with HCAECs pre-treated with increased concentration of either EET.

## Discussion

In the current study, it was shown that increased baseline serum levels of specific LOX- or CYP-catalyzed AA metabolites were likely associated with future development of AMI in a cohort of stable CAD patients with PCI. Further, serum levels of these specific HETEs, except for 11,12-EET, were positively correlated to that of TNF-α and NT-pro-BNP but not to hs-CRP or IL 6 or others. Accordingly, a series of specific serum oxylipins with significant prognostic impacts on clinical outcomes in stable CAD patients were identified. While preliminary and in need of further confirmation, our findings did provide a novel explanation of the potential role of serum oxylipins, independent of PUFA, for secondary prevention in clinical CAD.

Previous reports on omega-3 or omega-6 PUFA supplements for cardiovascular health are conflicting^10–16^. One meta-analysis revealed that replacement of SFA with LA reduced the CAD risk^10^. However, replacement of saturated fat by LA in men who had suffered a recent coronary event showed no benefit and possible harm in the Sydney Diet Heart Study and updated meta-analysis^11^. Inverse association with cardiovascular disease incidence with both dietary and circulating EPA and DHA, but not alpha-linolenic acid or omega-6 PUFAs, were shown in the Multi-Ethnic Study of Atherosclerosis^12^. One recent meta-analysis revealed no evidence that increasing omega-6 fats decreases cardiovascular outcomes, except for AMI^13^. Another recent meta-analysis revealed that increasing EPA and DHA have no significant impact on cardiovascular health or mortality^14^. However, the latest clinical trials revealed different results with high intake of omega-3 PUFAs and cardiovascular events. One clinical trial revealed that omega-3 PUFAs (icosapent ethyl) lower cardiovascular events^15^, whereas, another clinical trial revealed that supplementation with omega-3 PUFAs (marine omega-3 fatty acids) does not reduce the incidence of major cardiovascular events or cancer^16^. This inconsistent data may be due to the complexity of the PUFA metabolites.

PUFAs can be catalyzed into a series of oxylipins. Among them, LOXs and CYPs are a series of iron-containing enzymes that metabolize AAs to form a spectrum of biologically active products that might play different biological or pathological roles^28^. In the present study, elevated LOX-catalyzed AA metabolites (8-HETE, 9-HETE, 11-HETE, 12-HETE, and 15-HETE) could be linked to subsequent AMI in stable CAD patients. Some previous studies concerning LOX-catalyzed AA metabolites and atherosclerosis have been carried out. 12-HETE is associated with chemotaxis and regulation of leukocyte adherence and, therefore, contributes to vascular inflammation and atherosclerosis^29^. It has been suggested that the 12-LOX pathway increases the susceptibility of diabetics to atherosclerosis^30^. Additionally, it was shown that the expression of 15-HETE is increased in ischemic heart tissue^31^. The expression of arachidonate 15-LOX is also increased in ischemic human heart biopsy specimens^32^. It was then proposed that increased arachidonate 15-LOX expression in the heart under ischemic conditions may increase 15-HETE production, potentially causing thrombosis. On the other hand, a previous animal study analyzing the oxylipin profiles in the plaque found that the AA metabolite produced by the COX pathway, 6-keto-PGF1α, is most abundant, preceding the LA metabolites 9-HODE, 13-HODE and 9, 12,13-TriHOME, and the AA-derivatives 11-HETE and 12-HETE^33^. Furthermore, the most abundant plasma oxylipins are 11-HETE, 13-HODE, and 9-HODE^33^. Zu *et al*. reported significant increase of plasma levels of 8-HETE, 11-HETE, 12-HETE, and 15-HETE in patients with acute coronary syndrome compared to the control subjects, and 5-HETE whereas 9-HETE were significantly increased in patients with future major adverse cardiac events compared to the control^34^. These findings, together with ours, support the notion that LOX-catalyzed AA metabolites might be involved in the development of clinical cardiovascular events including AMI.

Some previous studies have shown that 20-HETE is important in the regulation of vascular function and is elevated in patients with hypertension and stroke^35^. One study indicated that higher circulating 20-HETE levels are associated with lower brachial artery flow-mediated dilation and higher circulating levels of cellular adhesion molecules are found in stable CAD patients which suggests that heightened CYP ω-hydroxylase activity might increase the stable CAD patients’ propensity to the development of endothelial dysfunction and vascular inflammation^36^. In the current study, baseline serum levels of 20-HETE as well as other specific CYP-catalyzed AA metabolites such as 19-HETE, 5,6-EET, 8,9-EET, 11,12-EET, and 14,15-EET could be increased in CAD patients with subsequent AMI compared to the patients without future cardiovascular events. Our findings further indicated the novel connections of 20-HETE with the onset of AMI.

EETs are 20-carbon metabolites of AA. CYP2J2 is an AA epoxygenase required for the formation of 5,6-EET, 8,9-EET, 11,12-EET, and 14,15-EET^37^. Genetic variation in CYP2J2 may be associated with increased risk of AMI^38–40^; however, the findings were not consistent^41^. Liu *et al*. reported that hypertension, diabetes, smoking, and CYP2J2 gene polymorphism were associated with early-onset AMI (<45 years) in Taiwan^38^. In Liu’s study, patients with early-onset AMI had more CYP2J2*7 GT + TT genotype. Furthermore, the CYP2J2*7 T allele carriers had significantly lower EET metabolites than carriers with GG allele (*P* < 0.05). In our study, however, the patients with higher baseline EET levels were associated with subsequent AMI. There are several potential mechanisms for the differences between the findings of Liu and colleagues and our findings. Firstly, the mean ages of our patients were older than that in the previous study. Secondly, both the subjects and controls were stable CAD patients in the current study, which were different from that in the previous study. Thirdly, in the current study, all the study subjects and control patients were prospectively followed up. Blood sampling was conducted at baseline much early before the events of AMI, suggesting the time sequence for potential causal relationship of baseline EET levels with subsequent AMI. There was no such data in the previous study. Finally, while the sample sizes were small, the clinical events such as AMI were clearly defined and the baseline characteristics were well matched in the study subjects with the control patients, suggesting the relatively homogeneous study cohorts in the current study. However, whether our observation of a high association between EET levels and subsequent AMI is due to a special or unique CYP gene (or LOX gene) polymorphism in Taiwanese patients is not clear, which warrants further investigation.

Interestingly, in the current study, the baseline AA metabolites, EETs or HETEs, were correlated with TNF-α in all the study subjects as well as control patients. While TNF-α is related to chronic inflammation, some previous studies have shown the pro-inflammatory property of HETEs^29,42^. In a group of 42 obese men and women, the concentrations of 12-HETE and 5-HETE are significantly increased in subjects with obesity and low-grade inflammation. After weight reduction, the levels of 12-HETE, 5-HETE, and TNF-α are significantly lowered^42^. However, there are also studies that reported the anti-inflammatory effects of AA-derived metabolites, mainly EETs^43–46^. One human study demonstrated that patients who are poor metabolizers of CYP2C19 have significantly lower EET levels and higher hs-CRP levels than their counterparts in 81 patients with microvascular angina. It was concluded that the decline in EET may induce chronic inflammation and affect coronary microvascular dysfunction^43^. Other studies had shown the anti-inflammatory property of some specific oxylipins (EETs or HETEs) through inhibition of the TNF-α pathway *in vitro* and *in vivo*^44–46^. In the current study, the 11 AA metabolites, either EETs or HETEs, were highly correlated with pro-inflammatory TNF-α, suggesting that AA-derived metabolites were associated with increased inflammation, which might be attributed in part to the development of AMI in the subject group of patients who had a potential for increased systemic inflammation. Moreover, our new findings indicate that pretreatment with 5,6-EET or 14,15-EET significantly increased the adhesiveness of HCAECs to THP-1 cells. Since previous studies had shown that increased cell adhesion is a sign of pro-inflammation and early phase of atherogenesis^47^, the findings did suggest the potential link between oxylipin EETs and vascular inflammation as well as atherogenesis.

In the current study, the baseline AA metabolites, EETs or HETEs, were also correlated with NT-pro-BNP. NT-pro-BNP is a cardiac biomarker that is effective in predicting the outcomes of acute coronary syndrome and heart failure^48^. However, some studies had reported the cardio-protective effects of AA-derived metabolites^49,50^. One animal study demonstrated that intracoronary infusion of 11,12-EET and 14,15-EET reduces infarct size in dogs compared to the control^49^. Another animal study suggested that EETs alleviate ethanol-induced myocardial dysfunction^50^. Further study is needed to clarify their role in the cardiac function of stable CAD patients.

Although the levels of TNF-α, NT-pro-BNP, and other biomarkers were similar between the groups, which might be due to small sample size, we found that circulating HETEs and EETs levels were different between the groups. These findings suggest that these AA-derived metabolites might be more sensitive as the potential biomarkers for prediction of future AMI in stable CAD patients. Further study may be required to elucidate the potential causal relationship of HETEs and EETs with TNF-α, NT-pro-BNP, or other inflammatory biomarkers either *in vitro* or *in vivo* or both.

In our study, all the patients encountering new-onset AMI during the 2-year follow-up period were selected from a secondary prevention cohort consisting of 2,239 stable CAD patients. We determined the baseline serum oxylipin profiles and followed up on the subsequent development of AMI. Previous studies were either conducted by cross-sectional design or lacked clinical outcomes^36,51^. For example, Schuck *et al*. reported that 20-HETE have an inverse association with brachial artery flow-mediated dilation^36^. Theken *et al*. reported that CAD patients, both obese and non-obese, have significantly higher plasma EETs (*P* < 0.01) and epoxide:diol ratios (*P* < 0.01) but not 20-HETE levels when compared to healthy volunteers^51^. Notably, we found for the first time that both circulating HETEs and EETs levels were higher in stable CAD patients with subsequent AMI.

It is well documented that omega-3/omega-6 fatty acid in diet may affect the level of the metabolites of interest in human blood. However, we collected the diet records of the control and subject groups of patients in the cohort and observed no special foods/supplements taken in the two groups which could contribute to change of PUFAs levels in patient’s sera. Moreover, we did not observe any significant difference of the level of LA and AA, the substrates of oxylipins in the two groups, either. Therefore, directly correlating the level of dietary fatty acids and oxylipins in circulating blood may not be practical because metabolism through gut microbiota in individual patients might result in high discrepancies in the oxidized fatty acid end-product/metabolites, including oxylipins.

### Study limitations

This study’s limitations include, for one thing, its small sample sizes. Further studies that include larger sample sizes are required to validate the current findings. Second, to make sure that the control patients may not suffer from any new-onset cardiovascular events including AMI in a sufficient follow-up period, they should live well over a significantly longer follow-up period than the study subjects did. Therefore, the follow-up durations were different between the subject and control groups. The Kaplan-Meier analysis and Cox proportional hazard regression were then performed to confirm the data of initial analyses. The findings are consistent. Third, the study enrolled only ethnic Chinese patients. Further studies may validate the results in other populations.

## Conclusion

Stable CAD patients who encountered subsequent AMI had higher baseline levels of specific AA metabolites when compared to those patients without subsequent cardiovascular events. These specific AA-derived oxylipins could be correlated to serum TNF-α and NT-pro-BNP levels at baseline. Further, 5,6-EET and 14,15-EET pre-treated HCAECs enhanced the adhesion of THP-1 cells, suggesting the EETs are inflammatory and play a role in the development of AMI in patients. While this is the first study to report that not only EETs but also HETEs were increased in stable CAD patients with subsequent cardiovascular events, future investigation is needed to clarify whether these oxylipins directly contribute to the development of clinical cardiovascular events such as AMI in patients with stable CAD.

## Supplementary information

Supplemental Tables.
