# Supplementary material for: Association of Arachidonic Acid-derived Lipid Mediators with Subsequent Onset of Acute Myocardial Infarction in Patients with Coronary Artery Disease
Source: Sci Rep. 2020 May 15;10:8105. doi: 10.1038/s41598-020-65014-z (PMC7229015; doi:10.1038/s41598-020-65014-z)
Supplement: Supplementary file 1 — Supplemental Tables. [file 41598_2020_65014_MOESM1_ESM.docx]

Association of Arachidonic Acid-derived Lipid Mediators with Subsequent Onset of Acute Myocardial Infarction in Patients with Coronary Artery Disease

Chin-Chou Huang^1,2,3,4^, Meng-Ting Chang^5,6^, Hsin-Bang Leu^2,3,7,8^, Wei-Hsian Yin^9^, Wei-Kung Tseng^10^, Yen-Wen Wu^11^, Tsung-Hsien Lin^12^, Hung-I Yeh^13^, Kuan-Cheng Chang^14^, Ji-Hung Wang^15^, Chau-Chung Wu^16^, Lie-Fen Shyur^5,6,17*^, and Jaw-Wen Chen^2,3,4,7,18*^

^1^Department of Medical Education, Taipei Veterans General Hospital, Taipei, Taiwan.

^2^Division of Cardiology, Department of Medicine, Taipei Veterans General Hospital, Taipei, Taiwan.

^3^Cardiovascular Research Center, National Yang-Ming University, Taipei, Taiwan.

^4^Institute of Pharmacology, National Yang-Ming University, Taipei, Taiwan.

^5^Agricultural Biotechnology Research Center, Academia Sinica, Taipei, Taiwan.

^6^Department of Biochemical Science and Technology, College of Life Science, National Taiwan University, Taipei, Taiwan.

^7^Healthcare and Service Center, Taipei Veterans General Hospital, Taipei, Taiwan.

^8^Institute of Clinical Medicine, National Yang-Ming University, Taipei, Taiwan.

^9^Division of Cardiology, Heart Center, Cheng-Hsin General Hospital, and School of Medicine, National Yang-Ming University, Taipei, Taiwan.

^10^Department of Medical Imaging and Radiological Sciences, I-Shou University and Division of Cardiology, Department of Internal Medicine, E-Da Hospital, Kaohsiung, Taiwan.

^11^Cardiology Division of Cardiovascular Medical Center and Department of Nuclear Medicine, Far Eastern Memorial Hospital, New Taipei City, Taiwan; School of Medicine, National Yang-Ming University, Taipei, Taiwan.

^12^Division of Cardiology, Department of Internal Medicine, Kaohsiung Medical University Hospital and Kaohsiung Medical University, Kaohsiung, Taiwan.

^13^Mackay Memorial Hospital, Mackay Medical College, New Taipei City, Taiwan.

^14^Division of Cardiology, Department of Internal Medicine, China Medical University Hospital, Taichung, Taiwan; Graduate Institute of Clinical Medical Science, China Medical University, Taichung, Taiwan.

^15^Department of Cardiology, Buddhist Tzu-Chi General Hospital, Tzu-Chi University, Hualien, Taiwan.

^16^Division of Cardiology, Department of Internal Medicine, National Taiwan University College of Medicine and Hospital, Taipei, Taiwan; Department of Primary Care Medicine, College of Medicine, National Taiwan University, Taipei, Taiwan.

^17^PhD Program in Translational Medicine, College of Medicine, Kaohsiung Medical University, Kaohsiung, Taiwan.

^18^Department of Medical Research, Taipei Veterans General Hospital, Taipei, Taiwan.

* Lie-Fen Shyur: lfshyur@ccvax.sinica.edu.tw

* Jaw-Wen Chen: jwchen@vghtpe.gov.tw

**Supplementary Tables**

**Supplemental Table 1. Metabolite standards representing oxylipins derived from linoleic acid (LA), arachidonic acid (AA), eicosapentaenoic acid (EPA), and docosahexaenoic acid (DHA) used to obtain the optimized MS/MS conditions and limits of quantification (LOQ)**

| **Analyte** | **Scan mode [M−H]^−^** | | **Collision energy (V)** | **LOQ (ppm)** |
| --- | --- | --- | --- | --- |
|  | **Precursor ion (*m/z*)** | **Product ion (*m/z*)** |  |  |
| LA | 279.0 | 279.0 | 5 | 0.0001 |
| 9-HODE | 295.2 | 171.0 | 20 |  |
| 9-oxoODE | 293.2 | 185.1 | 28 |  |
| 9,10,13-TriHOME | 329.2 | 171.1 | 32 |  |
| 9,12,13-TriHOME | 329.2 | 211.1 | 28 |  |
| 13-HODE | 295.2 | 195.0 | 20 |  |
| 13-oxoODE | 293.2 | 113.0 | 29 |  |
| 9,10-EpOME | 295.2 | 171.1 | 18 |  |
| 12,13-EpOME | 295.2 | 195.2 | 20 |  |
| 9,10-DHOME | 313.2 | 201.2 | 24 |  |
| 12,13-DHOME | 313.2 | 183.2 | 32 |  |
| AA | 303.0 | 259.0 | 18 | 0.001 |
| 5-HETE | 319.2 | 115.1 | 14 |  |
| 5-oxoETE | 317.2 | 273.2 | 20 |  |
| LTA_4_ | 311.0 | 183.0 | 40 |  |
| LTB_4_ | 335.2 | 195.1 | 23 |  |
| 8-HETE | 319.2 | 301.2 | 17 |  |
| 9-HETE | 319.2 | 123.1 | 20 |  |
| 11-HETE | 319.2 | 167.2 | 20 |  |
| 12-HETE | 319.2 | 179.2 | 20 |  |
| 15-HETE | 319.2 | 301.4 | 12 |  |
| 15-oxoETE | 317.2 | 113.1 | 24 |  |
| LXA_4_ | 351.1 | 115.0 | 30 |  |
| LXB_4_ | 351.0 | 217.0 | 24 |  |
| 19-HETE | 319.2 | 275.1 | 20 |  |
| 20-HETE | 319.2 | 275.2 | 14 |  |
| 5,6-EET | 319.2 | 191.0 | 20 |  |
| 8,9-EET | 319.2 | 123.0 | 20 |  |
| 11,12-EET | 319.2 | 167.0 | 16 |  |
| 14,15-EET | 319.2 | 219.3 | 20 |  |
| 5,6-DHET | 337.2  337.2 | 145.1  127.1 | 20 |  |
| 8,9-DHET |  |  | 30 |  |
| 11,12-DHET | 337.2 | 167.1 | 22 |  |
| 14,15-DHET | 337.2 | 207.1 | 20 |  |
| THF-diols | 353.2 | 167.1 | 32 |  |
| PGE_2_/PGD_2_ | 351.2 | 271.3 | 20 | 0.0001 |
| PGB_2_/PGJ_2_ | 333.2 | 235.3 | 28 |  |
| 15-deoxy-PGJ_2_ | 315.2 | 271.3 | 20 |  |
| 6-keto-PGF_1α_ | 383.4 | 187.0 | 34 |  |
| PGF_2α_ | 353.2 | 309.3 | 28 |  |
| TXB_2_ | 369.2 | 169.1 | 16 |  |
| EPA | 301.1 | 257.0 | 20 | 0.001 |
| 15-HEPE | 317.0 | 219.0 | 18 |  |
| DHA | 327.1 | 283.0 | 18 |  |
| 17-HDHA | 343.2 | 281.0 | 16 |  |
| 10,17-DiHDHA | 359.0 | 206.0 | 20 |  |
| Resolvin D1 | 375.3 | 121.0 | 35 |  |
| Maresin | 359.3 | 250.0 | 30 |  |

AA, arachidonic acid; DHA, docosahexaenoic acid; EPA, eicosapentaenoic acid; LA, linoleic acid; LOQ, limits of quantification.

**Supplemental Table 2. Dietary/food supplement records of the patients in the control and subject groups.**

|  | **Control group**  **(*n*=50)** | **Subject group**  **(*n*=25)** | ***P* value** |
| --- | --- | --- | --- |
| Food supplements, n (%) | 21 (42.0%) | 6 (24.0%) | 0.126 |
| ANKA (Red yeast rice), n (%) | 1 (2.0%) | 0 (0.0%) | 1.000 |
| Fish oil, n (%) | 3 (6.0%) | 0 (0.0%) | 0.546 |
| Phytosterol, n (%) | 0 (0.0%) | 0 (0.0%) | - |
| Cornmeal, n (%) | 18 (36.0%) | 6 (24.0%) | 0.294 |
| Ginko, n (%) | 2 (4.0%) | 0 (0.0%) | 0.500 |
| Natto, n (%) | 0 (0.0%) | 0 (0.0%) | - |
| Garlic essence, n (%) | 0 (0.0%) | 0 (0.0%) | - |
| Grape seed, n (%) | 0 (0.0%) | 1 (4.0%) | 0.333 |
| Grapefruit juice, n (%) | 0 (0.0%) | 0 (0.0%) | - |
| Shark cartilage, n (%) | 1 (2.0%) | 1 (4.0%) | 1.000 |
| Fur seal oil, n (%) | 0 (0.0%) | 0 (0.0%) | - |

**Supplemental Table 3 The optimal cut-off level and area under the receiver operating characteristic (ROC) curve of oxylipins**

| **Oxylipins** | **Cut-off level** | **Area under the ROC curve (95% CI)** | ***P* value** |
| --- | --- | --- | --- |
| 8-HETE | 30.92 ng/mL | 0.728 (0.595 – 0.862) | 0.002 |
| 9-HETE | 4.28 ng/mL | 0.694 (0.554 – 0.835) | 0.007 |
| 11-HETE | 4.77 ng/mL | 0.745 (0.610 – 0.880) | 0.001 |
| 12-HETE | 17.39 ng/mL | 0.738 (0.606 – 0.871) | 0.001 |
| 15-HETE | 32.37 ng/mL | 0.727 (0.595 – 0.860) | 0.002 |
| 19-HETE | 6.03 ng/mL | 0.747 (0.612 – 0.883) | 0.001 |
| 20-HETE | 11.34 ng/mL | 0.724 (0.586 – 0.863) | 0.002 |
| 5,6-EET | 34.42 ng/mL | 0.716 (0.586 – 0.846) | 0.003 |
| 8,9-EET | 24.50 ng/mL | 0.735 (0.599 – 0.871) | 0.001 |
| 11,12-EET | 74.26 ng/mL | 0.720 (0.582 – 0.859) | 0.002 |
| 14,15-EET | 44.53 ng/mL | 0.664 (0.521 – 0.807) | 0.024 |

CI, confidence interval; ROC, receiver operating characteristic.
